# THE OPTIMUM LEVEL OF MELD TO MINIMIZE THE MORTALITY ON LIVER TRANSPLANTATION WAITING LIST, AND LIVER TRANSPLANTED PATIENT IN SÃO PAULO STATE, BRAZIL

**DOI:** 10.1590/0102-672020230028e1746

**Published:** 2023-09-15

**Authors:** Eleazar CHAIB, João Luiz Erbs PESSOA, Claudio José STRUCHINER, Luiz Augusto Carneiro D’ALBUQUERQUE, Eduardo MASSAD

**Affiliations:** 1 Department of Gastroenterology, Faculty of Medicine, Universidade de São Paulo – São Paulo (SP), Brazil; 2 Statistics, Health Secretary – São Paulo (SP), Brazil; 3 Applied Mathematics, School of Applied Mathematics, Fundação Getulio Vargas – Rio de Janeiro (RJ), Brazil

**Keywords:** Transplantation, Liver Cirrhosis, Hospital Mortality, Checklist, Biological Models, Transplante, Cirrose Hepática, Mortalidade Hospitalar, Lista de Checagem, Modelos Biológicos

## Abstract

**BACKGROUND:**

After validation in multiple types of liver disease patients, the MELD score was adopted as a standard by which liver transplant candidates with end-stage liver disease were prioritized for organ allocation in the United States since 2002, and in Brazil, since 2006.

**AIMS:**

To analyze the mortality profile of patients on the liver transplant waiting list correlated to MELD score at the moment of transplantation.

**METHODS:**

This study used the data from the Secretary of Health of the São Paulo State, Brazil, which listed 22,522 patients, from 2006 (when MELD score was introduced in Brazil) until June 2009. Patients with acute hepatic failure and tumors were included as well. We also considered the mortality of both non-transplanted and transplanted patients as a function of the MELD score at presentation.

**RESULTS:**

Our model showed that the best MELD score for patients on the liver transplant waiting list associated to better results after liver transplantation was 26.

**CONCLUSIONS:**

We found that the best score for applying to liver transplant waiting list in the State of São Paulo was 26. This is the score that minimizes the mortality in both non-transplanted and liver transplanted patients.

## RESUMO

**RACIONAL:**

Desde 2002, após validação em múltiplos tipos de hepatopatias, o escore MELD foi adotado como padrão pelo qual os candidatos a transplante de fígado com doença hepática terminal têm sido priorizados para alocação de órgãos nos Estados Unidos, e em 2006 no Brasil.

**OBJETIVOS:**

Analisar a mortalidade de pacientes em lista de espera para transplante de fígado correlacionando com o MELD, no momento do transplante.

**MÉTODOS:**

Foram utilizados os dados da Secretaria de Saúde do Estado de São Paulo, Brasil, onde foram listados 22.522 pacientes, desde 2006 (quando o escore MELD foi introduzido no Brasil) até junho de 2009. Foram incluídos pacientes com falência hepática e tumores. A mortalidade de pacientes não transplantados e transplantados também foi considerada em função do escore MELD.

**RESULTADOS:**

Nosso modelo mostrou que o melhor valor do MELD, em pacientes em lista de espera para transplante e com melhores resultados, foi de 26. Este valor minimiza mortalidade em pacientes não transplantados bem comem pacientes na lista de espera para transplante de fígado.

**CONCLUSÕES:**

O escore MELD ótimo para entrar na lista de espera para transplante de fígado, no estado de São Paulo, é em torno de 26. Esse é o valor que minimiza a mortalidade tanto dos pacientes não transplantados em lista de espera, quanto dos submetidos à transplante de fígado.

## INTRODUCTION

The Model for End-Stage Liver Disease (MELD) score was originally developed and validated to assess the short-term prognosis of patients with cirrhosis undergoing the transjugular intrahepatic portosystemic shunt (TIPS) procedure^14^.

It was, thereafter, validated in multiple types of liver disease patients and adopted as a standard to prioritize organ allocation for liver transplant candidates with end-stage liver disease in the United States since 2002^2^, and in Brazil since 2006.

This scoring system utilizes three widely available laboratory values: total bilirubin (g/dL), creatinine (g/dL), and international normalized ratio (INR) of prothrombin time^15^.

The MELD system has an immediate impact on the liver transplant setting that leads to a reduction in the number of registrants on the waiting list for the first time ever, and a 15% reduction in mortality among thse patients^9-11^. Since the introduction of MELD as the primary allocation system, there has been an ongoing effort to improve this mathematical prioritization model^17^. 

Despite substantial advances in liver transplantation techniques, there is still a growing number of accumulating patients on the waiting list. The ultimate goal of the allocation system is the balance between justice and utility, which means optimizing the use of scarce donor organ resource and reducing liver transplant waiting list (LTWL) mortality, besides maximizing long-term outcome^12,13^.

Our aim was to analyze the mortality profile of patients on the LTWL, using a model to estimate the optimum level of MELD score for both patients, those entering the waiting list and those that will undergo liver transplantation surgery in São Paulo State, Brazil.

## METHODS

For this study, we utilized the data from the Secretary of Health of the São Paulo State, Brazil, which listed 22,522 patients, from 2006 (when MELD score was introduced in Brazil) until June 2009.

We began by assuming that patients with liver failure present themselves along a short time interval (T) with MELD scores (s) of variables magnitudes. In the liver tumor model case, we call this interval “&quot;at presentation&quot;”. During this interval we assumed that liver failure patients (N) are included in the transplantation waiting list, and that livers (F) are available to these patients. Note that, we employed the same notation as in the model for liver tumors presented in prior publication^8^.

**Figure 1:**
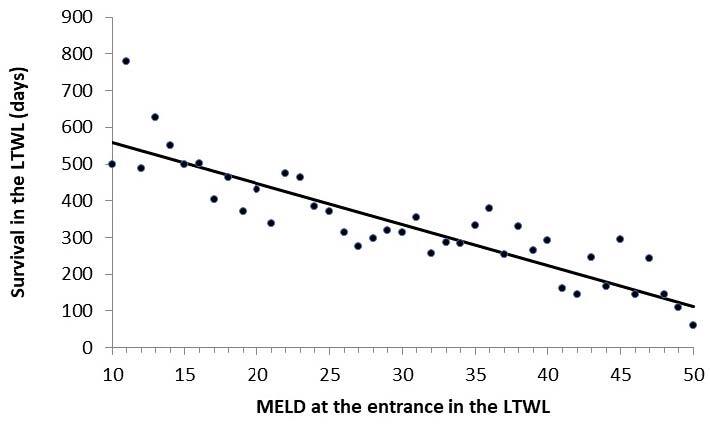
Survival in the liver transplant waiting list of non-transplanted patients as a function of model for end-stage liver disease at presentation.

MELD: model for end-stage liver disease; LTWL: liver transplant waiting list.

Were considered the mortality of non-transplanted and transplanted patients as a function of the MELD score at presentation. Figure 1 shows the probability density function of the MELD score of those 22,552 patients at presentation.

## RESULTS

Among the 22,552 patients listed in the LTWL from 2006 to 2019, a total of 6,121 were transplanted and 16,431 were not transplanted. Of the transplanted individuals, 2,401 died in the period, whereas 4,779 of the non-transplanted died in the list. This represents a total mortality of 39.2% for transplanted and 29% for non-transplanted patients.

We applied the Pearson’s chi-square test (χ^2^) to compare the significance (*p*) of the above difference, which resulted in χ^2^ = 195.667 with *p* ＜ 0.00001. This higher mortality rate among transplanted patients compared to non-transplanted patients on the list requires further investigation and this is the reason this analysis is presented in the future perspective chapter.

**Figure 2:**
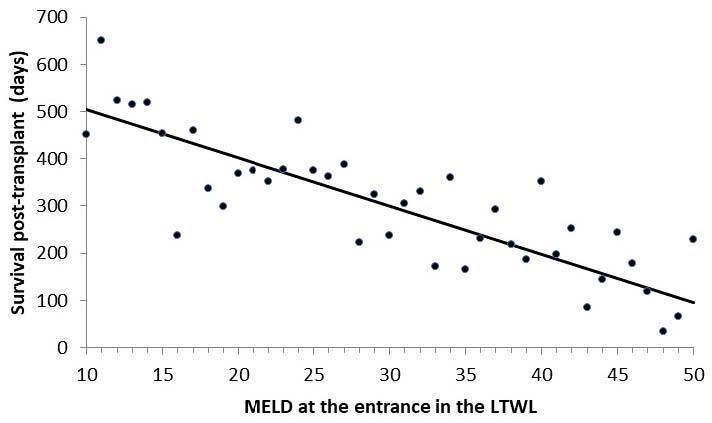
Survival of transplanted patients as a function of model for end-stage liver disease at presentation.

MELD: model for end-stage liver disease; LTWL: liver transplant waiting list.

The survival of both groups of patients along 16 years of analysis as a function of the MELD score at presentation for the non-transplanted and transplanted patients is shown in Figures 1 and 2, respectively. As can be observed in these figures, there is no difference between the two groups (Mann-Whitney U test: 11,777; p=0.56).

**Figure 3:**
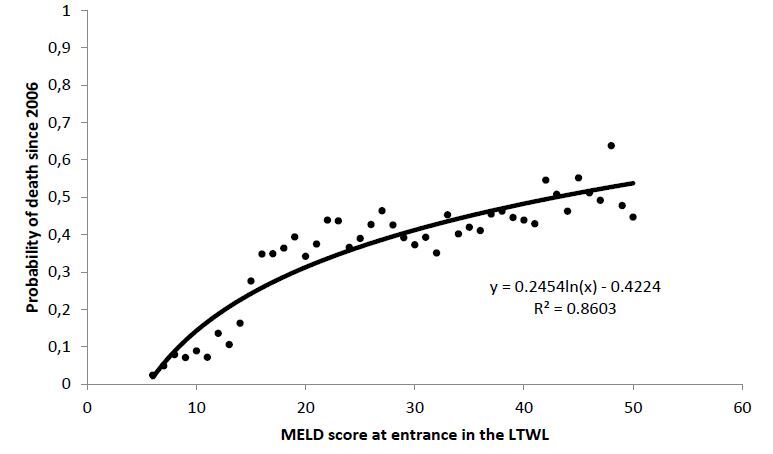
Death probability in the liver transplant waiting list of non-transplanted patients as a function of model for end-stage liver disease at presentation. Dots represent real data, solid line the average fitting and dotted lines the 95% confidence interval.

MELD: model for end-stage liver disease; LTWL: liver transplant waiting list.

**Figure 4:**
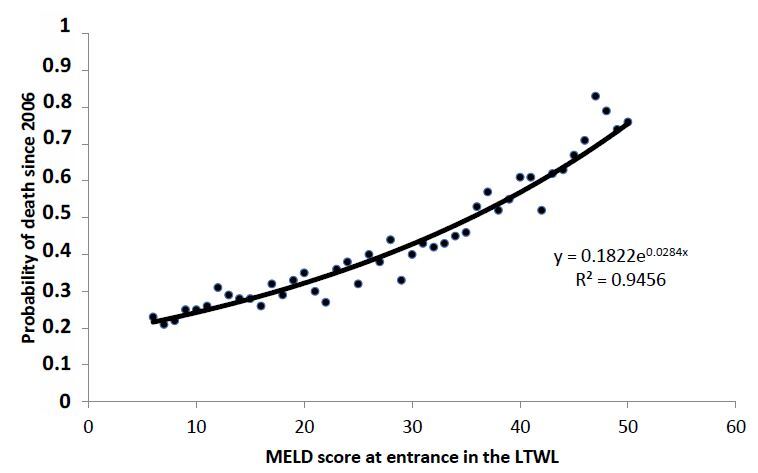
Death probability of transplanted patients as a function of model for end-stage liver disease at presentation. Dots represent real data, solid line the average fitting and dotted lines the 95% confidence interval.

MELD: model for end-stage liver disease; LTWL: liver transplant waiting list.

Subsequently, we calculated the probability of death for both groups along the 16 years of analysis as a function of MELD score at presentation. Figures 3 and 4 show the results for the non-transplanted and transplanted patients, respectively.

In this regard, the forms of the curves are entirely different from each other. The probability of death of non-transplanted patients growths logarithmically, whereas the probability of death of transplanted patients growths exponentially.

### Optimizing the meld score at entrance in the liver transplant waiting list

**Figure 5:**
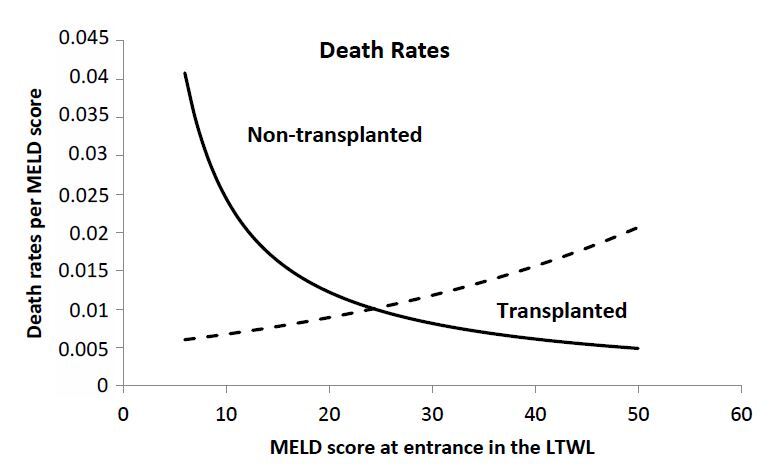
Mortality rates for transplanted and non-transplanted patients as a function of model for end-stage liver disease score at presentation. Continuous lines represent average and dotted lines the respective 95% confidence interval.

MELD: model for end-stage liver disease; LTWL: liver transplant waiting list.

**Figure 6:**
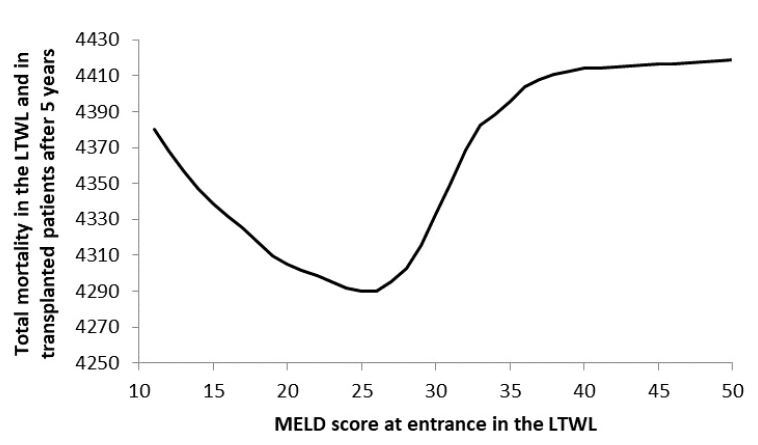
Total mortality in the liver transplant waiting list and in transplanted patients after 5 years as a function of model for end-stage liver disease score at presentation.

MELD: model for end-stage liver disease; LTWL: liver transplant waiting list.

As for the case of liver tumors^8^ the optimization model used is based on four assumptions, namely,

1. The mortality rates of non-transplanted *α*_*nt*_ and transplanted *α*_t_ liver failure patients are calculated from the actual mortality probabilities, according to the equations:
(1) ms=0/s
  and
(2) ts=eδs
  Where e is the MELD score at presentation and α, δ and β are the parameters obtained from the fitting of the Figures 3 and 4. Equations (1) and (2) assume that MELD scores increase with time, and so do the mortality rates. Equations (1) and (2) are illustrated in Figure 5, in which the mortality rates for both the transplanted and non-transplanted patients are presented as a function of the MELD score at presentation.
  The probability of surviving after *T* years for non-transplanted and transplanted patients, π_nt_(*s*) and π_t_(*s*), respectively, as a function of their MELD score *s* at the time individuals are included in the transplantation program, is given by:
(3) $$\text{nts}=\exp f_{\left(\right)}(-\text{ntT})$$
  and
(4) $$\text{ts}=\exp f_{\left(\right)}(-\text{tT})$$
Equations (3) and (4) result in survival probabilities after *T* years that are in agreement with the real data, as shown in Figure 1. They were used to calculate the forms and parameters of equations (1) and (2).
2. The mortality of both transplanted and non-transplanted patients is a monotonically increasing function of MELD score at presentation, as shown in Figures 3 and 4 (MELD score is, therefore, taken as an indication of gravity).
3. The number of available livers to be grafted, *F*, is limited and always less than the total number of liver failure patients, *N*, who have transplantation indication.
4. Finally, the MELD score, *s*, at the time individuals are included in the transplantation program, is distributed for the liver failure population according to an exponential distribution, according to the equation:
(5) fs,λ=eλs
  Where λ is the *rate parameter* of the distribution. This implies that in a liver failure population, many individuals have MELD scores of small magnitudes and few individuals have scores of large magnitudes. Again, this distribution of MELD score is performed at the time the patients enter the transplantation program. The cumulative distribution function (CDF) is given by:
(6) Fs,λ=0sesdt=1-eλs
Equation (6) means the probability that a given liver failure patient has MELD score equal or less than *s*.
  From the model of Chaib etal.^8^, we obtain the number of non-transplanted patients with MELD score greater than score *s*_0_ at presentation as:
(7) Ns0psds=Ns0esds
  and, among those, the survivors after time *T* are:
(8) Ns0eλsentsTds
  Hence, the total number of survivors after time *T* who were not transplanted is:
(9) NTS=N0s01-FNe-λse-ntsTds+Ns0e-λse-ntsTds
  Therefore, the total survival is obtained by adding equations (8) and (9):
(10) Survivors=F0s0e-λsetsTds+NTS
  Finally, the total mortality is given by:
(11) Ms0=N-Survivors
  To calculate the optimal transplantation strategy, we now determine the MELD score that can be transplanted and find both *s* and min[*M*(*s*)]. The result can be seen in the Figure 6.
  Note that the optimum MELD score to enter the LTWL is around 26. This is the value that minimizes mortality of both non-transplanted patients on the LTWL and transplanted patients.

## DISCUSSION

This paper applied a model originally designed to optimize liver transplantation in liver tumors patients^3^. The model provides a mathematical framework upon which an optimal strategy for organ allocation can be planned considering the MELD scores of patients in the LTWL.

The increased mortality of patients awaiting liver transplantation and the scarcity of donors’ organs induced efforts to improve allocation criteria for liver transplant candidates. The introduction of the MELD system in the United States for graft allocation resulted in a 3.5% reduction in the waiting list mortality, whereas the early-stage survival of liver transplant recipients remained unchanged, despite the more serious selection of ill patients for transplantation^10,11^.

Although MELD eliminates subjective assessments and shows accuracy in predicting the outcome in patients with decompensated cirrhosis, it has several limitations^16,17^. One of the limitations of the MELD score is that its components were found to independently and individually predict death on the waiting list^18^.

The major reason for implementing MELD was to decrease the number of deaths of the waiting list patients, providing each patient with an identical probability of receiving a transplant at presumed fixed condition levels.

Previously, priority was determined by a more complex system, in which the waiting list time and patient condition, classified in semiquantitative way, were linked (the presence of encephalopathy and ascites as well as the waiting time and patient location). It was established as an ultimate goal, to end the privilege of selecting the candidate on a clinical basis, considering various parameters such as the primary disease, degree of residual liver function, extrahepatic involvement, waiting list time, and donor-related risk, which was once a prerogative of the transplant surgeon.

The implementation of the new liver allocation system in our state, MELD (2006), has required a change in the disease severity score. In the pre-MELD era, the number of liver transplants increased 1,86-fold^7^; however, the number of patients on the LTWL increased 3,44-fold^5,6^ and the number of deaths of the waiting list patients increased 2,06-fold. This fact is reflected by the significant increase of the median MELD score at the time of liver transplant as well as by decreased median waiting time. We found that the median time on the waiting list decreased only for the patients who were submitted to liver transplant, whereas a significant proportion of patients with lower MELD scores were likely to have much longer waiting times.

After the implementation of MELD, we observed that the number of liver transplants increased 1.43-fold from 2006 to 2012; the number of patients on the LTWL was slightly reduced 0.95-fold. The number of deaths was significantly reduced 2.02-fold.

Numerous studies have investigated, with varying results, the prognostic value of the MELD score for early and late post-transplant survival^1-4^.

At our hospital, the recipients with a MELD score of 20–29 received organs fulfilling at least one extended donor criterion significantly more frequently. For the present study, we applied the model originally designed to optimize liver transplantation in patients with liver tumors^3^. It provides a mathematical framework upon which an optimal strategy for organ allocation can be designed considering the MELD scores of patients in the LTWL. With this model, we developed an optimal MELD score to enter LTWL minimizing the total number of deaths, both in patients on the list and in those transplanted.
